# Role of Glutathione in Cancer Progression and Chemoresistance

**DOI:** 10.1155/2013/972913

**Published:** 2013-05-20

**Authors:** Nicola Traverso, Roberta Ricciarelli, Mariapaola Nitti, Barbara Marengo, Anna Lisa Furfaro, Maria Adelaide Pronzato, Umberto Maria Marinari, Cinzia Domenicotti

**Affiliations:** Department of Experimental Medicine, Section of General Pathology, Via LB Alberti 2, 16132 Genoa, Italy

## Abstract

Glutathione (GSH) plays an important role in a multitude of cellular processes, including cell differentiation, proliferation, and apoptosis, and disturbances in GSH homeostasis are involved in the etiology and progression of many human diseases including cancer. While GSH deficiency, or a decrease in the GSH/glutathione disulphide (GSSG) ratio, leads to an increased susceptibility to oxidative stress implicated in the progression of cancer, elevated GSH levels increase the antioxidant capacity and the resistance to oxidative stress as observed in many cancer cells. The present review highlights the role of GSH and related cytoprotective effects in the susceptibility to carcinogenesis and in the sensitivity of tumors to the cytotoxic effects of anticancer agents.

## 1. Introduction

Reactive oxygen species (ROS) are physiologically produced by aerobic cells [1] and their production increases under conditions of cell injury [2]. Physiological levels of ROS mediate crucial intracellular signaling pathways and are essential for cell survival. However, an excess of ROS formation generates cell damage and death. To prevent the irreversible cell damage, the increase of ROS induces an adaptive response, consisting in a compensatory upregulation of antioxidant systems, aimed to restore the redox homeostasis [3].

Oxidative stress has long been implicated in cancer development and progression [4], suggesting that antioxidant treatment may provide protection from cancer [5]. On other hand, prooxidant therapies, including ionizing radiation and chemotherapeutic agents, are widely used in clinics, based on the rationale that a further oxidative stimulus added to the constitutive oxidative stress in tumor cells should, in fact, cause the collapse of the antioxidant systems, leading to cell death [6]. However, this latter approach has provided unsatisfactory results in that many primary tumors overexpress antioxidant enzymes at very high levels, leading to a resistance of cancer cells to drug doses [7].

Among the enzymatic systems involved in the maintenance of the intracellular redox balance, a main role is played by GSH [8] that participates, not only in antioxidant defense systems, but also in many metabolic processes [9]. 

Elevated GSH levels are observed in various types of tumors, and this makes the neoplastic tissues more resistant to chemotherapy [10, 11]. Moreover, the content of GSH in some tumor cells is typically associated with higher levels of GSH-related enzymes, such as *γ*-glutamylcysteine ligase (GCL) and *γ*-glutamyl-transpeptidase (GGT) activities, as well as a higher expression of GSH-transporting export pumps [11, 12]. Therefore, it is not surprising that the GSH system has attracted the attention of pharmacologists as a possible target for medical intervention against cancer progression and chemoresistance. 

The main research in this field has been aimed at depleting GSH by a specific inhibition of GCL, a key enzyme of GSH biosynthesis. In this context, buthionine sulfoximine (BSO) is the most popular GSH-depleting agent studied in both preclinical and early clinical trials, but limitation on its availability has led to a search for alternatives [13, 14]. Recently, GSH analogues have been employed in order to sensitize tumors to cytotoxic effects of anticancer agents, by depleting GSH-related cytoprotective effects [15].

However, during the last decade, a new approach for the regulation of GSH-utilizing enzymes has emerged. It is also evident that many of the antioxidant enzymes are induced by GSH depletion at the transcriptional level which involves the binding of the nuclear factor (erythroid-derived 2)-like 2 (Nrf2) transcription factor to the antioxidant response element (ARE) in the promoter region of the genes encoding GCL and glutathione *S*-transferases [16].

## 2. GSH Biosynthesis

Glutathione (GSH) is a tripeptide formed by glutamic acid, cysteine, and glycine. The glutamic acid forms a particular gamma-peptic bond with cysteine by its gamma glutamyl group. Two forms of GSH are possible: the reduced form (GSH) which represents the majority of GSH, reaching millimolar concentration in the intracellular compartment, and the oxidized form (GSSG) that is estimated to be less than 1% of the total GSH. Intracellularly, the majority of GSH is found in the cytosol (about 90%), while mitochondria contain nearly 10% and the endoplasmic reticulum contains a very small percentage [17].

The synthesis of GSH from its constituent amino acids involves two ATP-requiring enzymatic steps: (1) the first step is rate-limiting and catalyzed by GCL which is composed of two subunits: one catalytic (GCLC) and one modifier (GCLM):


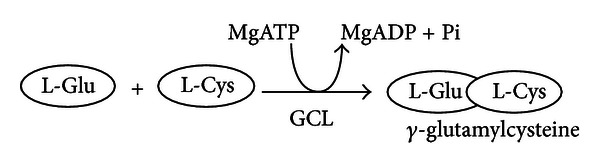


(2) The second step is catalyzed by GSH synthetase (GS) [19]:


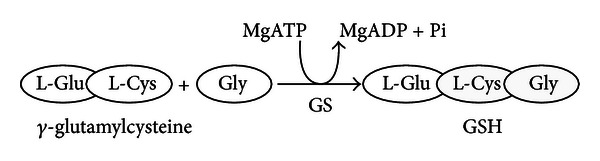


Although GS is generally thought not to be important in the regulation of GSH synthesis, accumulating evidence suggests that GS may play an important role, at least in certain tissues and/or under stressful conditions [20].

However, under normal physiological conditions, the rate of GSH synthesis is largely determined by two factors, that is, cysteine availability and GCL activity. Cysteine is normally derived from the diet, protein breakdown and in the liver, from methionine via transsulfuration (conversion of homocysteine to cysteine). Cysteine differs from other amino acids because its sulfhydryl form, cysteine, is predominant inside the cell whereas its disulfide form, cystine, is predominant outside the cell [21].

## 3. Glutathione Functions

The chemical structure of GSH determines its potential functions, and its broad distribution among all living organisms reflects its important biological role. A major function of GSH is the detoxification of xenobiotics and some endogenous compounds. These substances are electrophiles and form conjugates with GSH, either spontaneously or enzymatically, in reactions catalyzed by GSH-*S*-transferases (GST) [22]. Human GSTs are divided into two distinct family members: the membrane-bound microsomal and cytosolic family members. The conjugates formed are usually excreted in the bile, but can also undergo modification to mercapturic acid. 

Another important GSH function is the maintenance of the intracellular redox balance and the essential thiol status of proteins [21].

The reaction with the protein is as follows:

(1) $$\begin{array}{c} \text{Protein-SSG}+\text{GSH}⟷\text{Protein-SH}+\text{GSSG}. \end{array}$$

**Figure 1:**
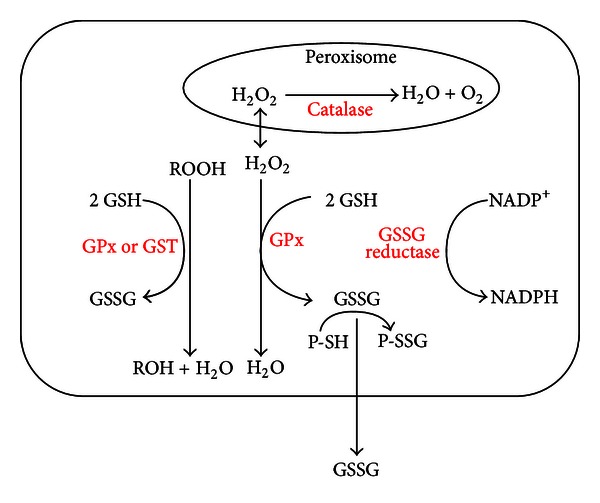
Antioxidant function of GSH. The hydrogen peroxide, produced during the aerobic metabolism, can be metabolized in the cytosol by GSH peroxidase (GPx) and catalase in peroxisomes. In order to prevent oxidative damage, the GSSG is reduced to GSH by GSSG reductase at the expense of NADPH, forming a redox cycle [17]. Organic peroxides can be reduced both by GPx and GSH-transferase (GST). In extreme conditions of oxidative stress, the ability of the cell to reduce GSSG to GSH may be less, inducing the accumulation of GSSG within the cytosol. In order to avoid a shift in the redox equilibrium, the GSSG can be actively transported out of the cell or react with protein sulfhydryl groups (PSH) and form mixed disulfides (PSSG).

The equilibrium of this reaction depends on the concentrations of GSH and GSSG. The reversible thiolation of proteins is known to regulate several metabolic processes including enzyme activity, transport activity, signal transduction and gene expression through redox-sensitive nuclear transcription factors such as AP-1, NF-kappaB (NF-kB) and p53 [21, 23]. In fact, DNA-binding activity of transcription factors often involves critical Cys residues, and the maintenance of these residues in a reduced form, at least in the nuclear compartment, is necessary [24]. AP-1 is a transcription factor related to tumor promotion [25], and its DNA-binding activity can be diminished if Cys-252 is oxidized [26]. Tumor suppressor p53, known as the “guardian of the genome,” contains 12 Cys residues in its amino acid sequence [27], and oxidation of some of these inhibits p53 function [28]. Moreover, GSH performs an antioxidant function (Figure 1).

**Figure 2:**
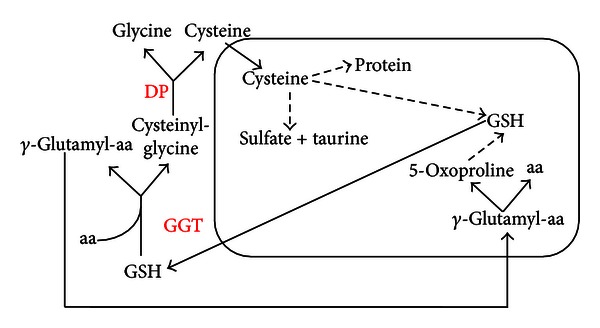
*γ*-Glutamyl cycle. In the *γ*-glutamyl cycle, GSH is released from the cell and the ectoenzyme GGT transfers the *γ*-glutamyl moiety of GSH to an amino acid (aa, the best acceptor being cysteine), forming *γ*-glutamyl-aa and cysteinyl-glycine. The *γ*-glutamyl-amino acid can then be transported back into the cell and once inside can be further metabolized to release the aa and 5-oxoproline, which can be converted to glutamate and used for GSH synthesis. Cysteinyl-glycine is broken down by dipeptidase (DP) to generate cysteine and glycine. Once inside the cell, the majority of cysteine is incorporated into GSH, some being incorporated into protein, and some degraded into sulfate and taurine [18].

In addition, storage of cysteine is one of the most important functions of GSH because cysteine is extremely unstable extracellularly and rapidly autooxidizes to cystine in a process that produces potentially toxic oxygen-free radicals [29]. The *γ*-glutamyl cycle allows GSH to be the main source of cysteine (Figure 2). 

## 4. Role of GSH in Regulating Cancer Development and Growth 

In many normal and malignant cells, increased GSH level is associated with a proliferative response and is essential for cell cycle progression [30, 31]. The molecular mechanism of how GSH modulates cell proliferation remains largely speculative. A key mechanism for GSH's role in DNA synthesis relates to the maintenance of reduced glutaredoxin or thioredoxin, which is required for the activity of ribonucleotide reductase, the rate-limiting enzyme in DNA synthesis [32].

Furthermore, in liver cancer and metastatic melanoma cells, GSH status is correlated with growth [33–35] and it has also been demonstrated that a direct correlation between GSH levels associated with cellular proliferation and metastatic activity exists [33]. In fact, intrasplenic inoculation of B16 melanoma (B16M) cells into C57BL/6J syngenic mice induced metastatic foci formation by colonizing different organs. However, the number and size of metastases were much higher when B16M cells with high GSH content were inoculated *in vivo* [33]. A high percentage of tumor cells with high GSH content were able to survive in the presence of the nitrosative and oxidative stress, thereby representing the main task force in the metastatic invasion [36]. Therefore, it is plausible that maintenance of high intracellular levels of GSH could be critical for the extravascular growth of metastatic cells. Moreover, maintenance of mitochondrial GSH homeostasis may be a limiting factor for the survival of metastatic cells in the immediate period following intra-sinusoidal arrest and interaction with activated vascular endothelial cells. Mitochondrial dysfunction is a common event in the mechanism leading to cell death [37], and, recently, it has been found to be an essential step for the killing of non-small-cell lung (NSCLC) carcinomas which are resistant to conventional treatments [38]. Thus, the impairment of GSH uptake by mitochondria may be important to sensitize invasive cancer cells to prooxidant compounds capable of activating the cell death mechanism. 

As previously reported, GSH is effluxed by cells through GGT-mediated metabolism, allowing a “GSH-cycle” to take place, which is implicated in tumor development [39]. In fact, GGT-positive foci were found in animals exposed to prooxidant carcinogens, suggesting the hypothesis of GGT as an early marker of neoplastic transformation [40, 41]. Moreover, increased levels of GGT have been observed in cancers of the ovaries [42], colon [43], liver [44], melanoma [45], and leukemias [46]. In studies on melanoma cells *in vitro *and *in vivo*, elevated GGT activity has been found to accompany an increased invasive growth [45, 47, 48], and a positive correlation has been described between GGT expression and unfavourable prognostic signs in human breast cancer [49].

The prooxidant activity of GGT has also recently been shown to promote the iron-dependent oxidative damage of DNA in GGT-transfected melanoma cells, thus potentially contributing to genomic instability and an increased mutation risk in cancer cells [50]. GGT/GSH-dependent prooxidant reactions has been shown to exert an antiproliferative action in ovarian cancer cells [51], while other studies in U937 lymphoma cells have shown that basal GGT-dependent production of hydrogen peroxide can represent an antiapoptotic signal [52]. The modulatory effects of GGT-mediated prooxidant reactions could contribute to the resistance phenotype of GGT-expressing cancer cells by regulating both signal transduction pathways involved in proliferation/apoptosis balance, as well as by inducing protective adaptations in the pool of intracellular antioxidants.

## 5. GSH Depletion as an Experimental Approach to Sensitize Tumor Cells to Therapy

Cancer cell lines containing low GSH levels have been demonstrated to be much more sensitive than control cells to the effect of irradiation [18]. In fact, GSH depletion obtained by BSO, the irreversible inhibitor of GCL, is the most frequently used approach and it is associated with many chemotherapeutic agents [53–57]. However, molecular signaling of BSO-induced apoptosis is poorly understood, and, recently, it has been demonstrated that in different leukaemia and lymphoma cells, the death receptor-mediated apoptotic pathway, induced by arsenic trioxide plus BSO, is triggered via JNK activation [58]. Moreover, in neuroblastoma cells susceptible to BSO treatment, DNA damage and apoptosis was triggered via PKC-*δ* activation and ROS production [59, 60]. 

In fact, BSO in combination with melphalan [14, 61], is currently undergoing clinical evaluation in children with neuroblastoma (NCT00002730; NCT00005835) and in patients with persistent or recurrent stage III malignant melanoma (NCT00661336). Recently, it has been demonstrated that a combination of azathioprine with BSO is useful for localized treatment of human hepatocellular carcinoma [62].

Therefore, BSO clinical use is restricted by its short half-life, with the consequent need for prolonged infusions resulting in its nonselective effect of GSH depletion on both normal and malignant cells [63]. 

## 6. Role of GSH in Chemoresistance

The increase in GSH levels, GCL activity and GCLC gene transcription is associated with drug resistance in tumor cells [64, 65]. 

The increase in GSH is a major contributing factor to drug resistance by binding to or reacting with, drugs, interacting with ROS, preventing damage to proteins or DNA, or by participating in DNA repair processes. In melanoma cells, GSH depletion and GGT inhibition significantly increased cytotoxicity via oxidative stress [66]. In addition, it has been demonstrated that GGT-overexpressing cells were more resistant to hydrogen peroxide [67] and chemotherapics, such as doxorubicin [68], cisplatin [64], and 5-fluorouracil [69].

Moreover, it has been found that the human multidrug resistance protein (MRP), a member of the superfamily of ATP-binding cassette membrane transporters, can lead to resistance to multiple classes of chemotherapeutic agents [70, 71]. Several studies have shown coordinated overexpression of GCLC and MRP in drug-resistant tumor cell lines, in human colorectal tumors and in human lung cancer specimens after platinum exposure [70, 71]. 

Three mechanisms have been proposed for the role of GSH in regulating cisplatin (CDDP) resistance: (i) GSH may serve as a cofactor in facilitating MRP2-mediated CDDP efflux in mammalian cells; (ii) GSH may serve as a redox-regulating cytoprotector based on the observations that many CDDP-resistant cells overexpress GSH and *γ*-GCS; and (iii) GSH may function as a copper (Cu) chelator.

Moreover, overexpression of specific GSTs can also affect chemoresistance, whereas polymorphisms that decrease GST activity are associated with a high risk of developing cancer [72]. An elevated expression of GSTs, combined with high GSH levels, can increase the rate of conjugation and detoxification of chemotherapy agents, thus reducing their effectiveness [73]. In addition to the transferase function, GSTs have been shown to form protein-protein interactions with members of the mitogen activated protein (MAP) kinases. By interacting directly with MAPKs, including c-Jun N-terminal kinase 1 (JNK1) and apoptosis signal-regulating kinase 1 (ASK1), GSTs bind the ligand in a complex structure, preventing interactions with their downstream targets [74]. Many anticancer agents induce apoptosis via activation of MAP kinases, in particular JNK and p38 [75, 76]. This novel, nonenzymatic role for GSTs has direct relevance to the GST overexpressing phenotypes of many drug-resistant tumors. As an endogenous switch for the control of intracellular signaling pathways, an elevated expression of GST can alter the balance of kinases during drug treatment, thereby causing a potential selective advantage for tumor growth.

The promoter regions encoding GSTs and *γ*GCL possess binding sites for transcriptional regulators such as NF-kB, AP-1, AP-2, and the Nrf2/Kelch-like ECH-associated protein 1 (Keap1) system. After exposure to oxidative stimuli, Nrf2 dissociates from Keap1, its negative regulator, and translocates into the nucleus where it heterodimerizes with small Maf proteins [77] and binds to antioxidant responsive element (ARE) sequences, triggering a cytoprotective adaptative response. This response is characterised by upregulation of several cytoprotective and detoxification genes, including ferritin, GSH-*S*-reductase (GSR), GST, GCLM, and GCLC, phase-I drug oxidation enzyme NAD(P)H:quinone oxidoreductase 1 (NQO1), MRP, and heme oxygenase-1 (HO-1) [78, 79]. 

However, in numerous types of cancer, Nrf2 is upregulated and takes on a protumoral identity since the above-cited cytoprotective genes, not only give tumors an advantage, but also lead to drug resistance [80–82].

To date, numerous mutations have been found of both Keap1 and Nrf2 in various human cancers resulting in the constitutive expression of prosurvival genes. Most of Nrf2 somatic mutations have led to the impairment of their recognition site for Keap1, which then has led to the continuous activation of Nrf2. Moreover, it has been observed that the prognosis of patients with either Nrf2 or Keap1 mutations is much lower than patients with no mutation [83].

Among Nrf2-regulated genes, HO-1 is the most well known as a stress protein that can have both antioxidative and anti-inflammatory effects [84]. It catalyzes the rate-limiting step in the catabolism of the prooxidant heme to carbon monoxide, biliverdin, and free iron [85]. 

Recent experimental evidence has shown the involvement of HO-1 in cancer cell biology. On one hand, HO-1 protects healthy cells from transformation into neoplastic cells by counteracting ROS-mediated carcinogenesis and, on the other hand, HO-1 protects cancer cells, enhancing their survival and their resistance to anticancer treatment [86]. In addition, high levels of HO-1 have been observed in various human solid tumors, such as renal [87], prostatic [88], and pancreatic cancers [89]. Moreover, HO-1 expression in tumor cells can be further increased by anticancer treatments (chemo-, radio-, and photodynamic therapy) [90], and it has been hypothesized that HO-1 and its products may have an important role in the development of a resistant phenotype. In this context, it has been recently demonstrated that BSO and/or the inhibition of the Nrf2/HO-1 axis is able to increase the sensitivity of neuroblastoma cells to etoposide [91, 92].

## 7. Therapeutic Potential of GSH and GSH-Modulating Agents

The modulation of the GSH-based antioxidant redox system (GRS), the major determinant of the cellular redox status [93], might represent a promising therapeutic strategy for overcoming cancer cell progression and chemoresistance. 

However, GSH itself cannot be administered clinically with any effect, and for this reason, a variety of precursors or chemically modified analogues have been generated in order to mimic glutathione's various physiological or pharmacological effects. *N*-acetylcysteine (NAC; Mucomyst) represents the earlier GSH analogue, and YM737, a monoester of GSH, recently discovered, has been favourably compared to NAC [94]. Another GSH analogue approach is cysteine-substituted *S*-nitrosoglutathione [95]. 

Since abundant levels of GST [96] have been identified in resistant tumors, GSH analogues, that differentially inhibit GST isoforms, have been developed. Telcyta (TLK-286) is a GSH analogue utilized in combination with cytotoxic chemotherapies such as platinum, taxanes, and anthracyclines in a variety of tumors with very high levels of glutathione *S*-transferase pi-1-gene (GST-P1-1) [97]. Telintra (TLK199) is another small molecule inhibitor of GST-P1-1 developed for the potential prevention of myelosuppression in blood diseases, namely, myelodysplastic syndrome [98]. After a phase 1 clinical trial [99] and a phase 2 using an oral formulation, TLK199 appeared to be well tolerated and with some efficacy in the myeloplastic syndrome treatment [100].

In addition, the implication of GGT activity in the resistance phenotype of cancer cells suggests a potential use of GGT inhibitors associated with chemotherapeutics in order to deplete intracellular GSH and/or to inhibit extracellular drug detoxification. Different GGT inhibitors are known [101–103], but, unfortunately, these molecules are toxic and cannot be used in humans. 

Moreover, drugs that target *S*-glutathionylation have direct anticancer effects since they act on a wide range of signalling pathways [57]. Among the agents that mediate their effects through *S*-glutathionylation, NOV-002 has been most extensively studied, with a phase III trial (NCT00347412) completed in advanced NSCLC [104], and data available from phase II trials in breast and ovarian cancers [105]. NOV-002 is a product containing oxidized glutathione that alter the GSH : GSSG ratio and induces *S*-glutathionylation [106]. NOV-002-induced *S*-glutathionylation has been shown to have inhibitory effects on proliferation, survival, and invasion of myeloid cell lines and significantly increased the efficacy of cyclophosphamide chemotherapy in a murine model of colon cancer [107].

In a randomized phase II trial, NOV-002 in combination with standard chemotherapy has shown promising effects in patients with stage IIIb/IV of NSCLC [108]. Positive results were also obtained from a phase II trial in patients with neoadjuvant breast cancer therapy [109].

Other therapeutic agents include phenolic antioxidants (*α*-napthoflavone, butylated hydroxyanisole, and *tert*-butyl hydroquinone), synthetic antioxidants (ethoxyquin, oltipraz, and phorbol esters), triterpenoid analogue (oleanolic acid derivatives, sesquiterpenes), and isothiocyanates (sulforaphane). Sulforaphane (SF) is the strongest natural inducer of Nrf2 and phase II detoxifying enzymes and it has a potent anticarcinogenic and chemopreventive effect by inducing apoptosis and cell cycle arrest [110]. 

On the other hand, the inhibition of Nrf2 signaling might be employed to enhance the sensitization of chemoresistant tumors to cytotoxic agents, and in this context, it has recently been reported that brusatol, a compound found in a plant extract, acts as inhibitor of this pathway and may exhibit therapeutic utility [111]. Another effective approach to increasing cancer cell sensitivity to chemotherapeutic drugs would be to silence both Nrf2 and Keap1 simultaneously [112]. Related to Nrf2, a potential target for redox chemotherapy is HO-1. HO-1 inhibitors, including zinc protoporphyrin and more soluble pegylated derivatives (PEG-ZnPP), have been successfully used to improve chemosensitization of cancer cells [113]. Moreover, HO-1 inhibitors administered intravenously, displayed cytotoxic activity in a murine hypoxic solid tumor model [114].

Moreover, disulfiram (DSF) does not cause depletion of total GSH, but shifts the ratio of GSH/GSSG towards the oxidized state. DSF induces apoptosis of human melanoma cells [115], and this apoptogenic effect has encouraged ongoing clinical phase I/II studies in human metastatic melanoma (NCT00256230). 

Arsenic trioxide (As_2_O_3_) is a prooxidant chemotherapeutic compound combined with agents that deplete cellular GSH [116]. As_2_O received FDA approval in 2000 for the treatment of nonacute promyelocytic leukemia and is used in patients who have relapsed or are refractory to first-line intervention using retinoid and anthracycline chemotherapy.

As_2_O inhibits GPx and mitochondrial respiratory function that leads to increased ROS leakage contributing to antileukemia activity. Another piece of evidence suggests that the irreversible inhibition of thioredoxin reductase is the key mechanism underlying As_2_O-induced breast cancer cell apoptosis [117]. Importantly, As_2_O_3_ sensitivity is associated with low levels of GSH in cancer cells and GSH depletion, obtained by BSO or ascorbate treatment, contributes to sensitizing cells toward apoptosis [118]. The potentiation of As_2_O chemotherapeutic efficacy using BSO was demonstrated in an orthotopic model of prostate cancer metastasis [119].

## 8. Conclusions

The modulation of cellular GSH is a double-edged sword, both sides of which have been exploited for potential therapeutic benefits [120]. Enhancing the capacity of GSH and its associated enzymes, in order to protect cells from redox-related changes or environmental toxins, represents a persistent aim in the search for cytoprotective strategies against cancer. On the contrary, the strategy of depleting GSH and GSH-related detoxification pathways is aimed at sensitizing cancer cells to chemotherapy, the so-called chemosensitization [121]. In this context, it has been reported that GSH and GSH enzyme-linked system may be a determining factor for the sensitivity of some tumors to various chemotherapeutic agents. In particular, GST is a relevant parameter for chemotherapy response, and it may be utilized as a useful biomarker for selecting tumors potentially responsive to chemotherapeutic regimens.

However, the attempts to deplete GSH have been limited by the nonselective effects of BSO and have stimulated the research of new GCL inhibitors.

Since it is well known that GSH depletion leads to the upregulation of antioxidant genes, many of which are under Nrf2 control and, that in several types of tumors, Nrf2 is constitutively activated [122, 123], a new and indirect approach for cancer therapy may be used to modulate the Nrf2-ARE pathway. Based on this, Nrf2 creates a new paradigm in cytoprotection, cancer prevention, and drug resistance. 

In summary, the involvement of GSH in the carcinogenesis and in the drug resistance of tumor cell is clear, but further studies, aimed at understanding the GSH-driven molecular pathways, might be crucial to design new therapeutic strategies to fight cancer progression and overcome chemoresistance.
